# Maternal incarceration increases the risk of self-harm but not suicide: a matched cohort study

**DOI:** 10.1017/S2045796023000264

**Published:** 2023-05-10

**Authors:** Craig Cumming, Megan F. Bell, Leonie Segal, Matthew J. Spittal, Stuart A. Kinner, Susan Dennison, Sharon Dawe, David B. Preen

**Affiliations:** 1 School of Population and Global Health, The University of Western Australia, Crawley, Australia; 2 Australian Centre for Precision Health, University of South Australia, Adelaide, Australia; 3 Melbourne School of Population and Global Health, The University of Melbourne, Parkville, Australia; 4 Centre for Adolescent Health, Murdoch Children’s Research Institute, Melbourne, Australia; 5 Justice Health Unit, Melbourne School of Population and Global Health, The University of Melbourne, Parkville, Australia; 6 Griffith Criminology Institute, Griffith University, Brisbane, Australia; 7 School of Population Health, Curtin University, Perth, Australia; 8 School of Criminology and Criminal Justice, Griffith University, Brisbane, Australia; 9 School of Applied Psychology, Griffith University, Brisbane, Australia; 10 The Hopkins Centre, Griffith University, Brisbane, Australia

**Keywords:** child abuse, mental health, self-injurious behaviour, social environment, suicide

## Abstract

**Aims:**

Children of incarcerated mothers are at increased risk of experiencing multiple adversity such as poverty, mental illness and contact with child protection services (CPS), including being taken into out of home care (OOHC). However, little is known about whether these children are at increased risk of suicide or self-harm compared to children not exposed to maternal incarceration or about the factors that may contribute to this. We aimed to investigate differences in the risk of suicide and self-harm between children exposed to maternal incarceration and those not exposed and examine how socio-demographic factors, maternal mental illness and CPS contact (with or without OOHC) may affect these outcomes.

**Methods:**

We used a retrospective matched cohort study design, comparing 7674 children exposed to maternal incarceration with 7674 non-exposed children. We used multivariable Cox proportional hazards regression to compare the risk of suicide and self-harm between exposed and non-exposed groups, controlling for geographical remoteness, CPS contact and maternal mental illness.

**Results:**

There was no significant difference in the rate of suicide (rate ratio [RR] = 1.49; 95% confidence interval [CI]: 0.78, 2.87) or risk of suicide (adjusted hazard ratio [aHR] = 0.92; 95% CI: 0.43, 1.96) between the two groups. However, the exposed group had a significantly higher rate of self-harm (RR = 2.83; 95% CI: 2.50, 3.21) and a significantly higher risk of self-harm (aHR = 1.74; 95% CI: 1.45, 2.09) compared to those non-exposed. CPS contact with or without OOHC was independently associated with an increased risk of self-harm for both groups.

**Conclusion:**

Children exposed to maternal incarceration are at an increased risk of self-harm and should be prioritized to receive targeted, multimodal support that continues after the mother’s release from prison. The association between CPS contact and self-harm warrants further research.

## Introduction

People who experience incarceration are exposed to multiple adversities, including high rates of health and social problems such as mental illness, substance use disorders (Abbott *et al.*, 2016; Fazel and Seewald, 2012), poverty and a history of child maltreatment and domestic violence (Friestad *et al.*, 2014; Wilson *et al.*, 2017). The prevalence of these adversities is particularly high among incarcerated women (Bartlett and Hollins, 2018). There is growing evidence that the stressors associated with incarceration not only directly impact the individual incarcerated but also directly and/or indirectly impact other family members, including children (Lee *et al.*, 2014; Turney, 2014; Wildeman *et al.*, 2012). These stressors are not confined to periods of incarceration. Children’s exposure to illegal or anti-social behaviour, including domestic violence, that occur around periods of parental incarceration (characterized as “packages” of risk [Giordano and Copp, 2015]) often contributes to intergenerational transmission of an increased risk of contact with the criminal justice system and social disadvantage for these children (Giordano *et al.*, 2019). The available evidence suggests that children of incarcerated parents are at an increased risk of mental illness (Lee *et al.*, 2013; Turney, 2014), physical health problems (Lee *et al.*, 2013), preventable mortality (Dowell *et al.*, 2018; Wildeman, 2012; Wildeman *et al.*, 2014a), placement into out of home care (OOHC) (Whitten *et al.*, 2021) and developmental issues such as learning disabilities, speech or other language problems, emotional vulnerability and behavioural or conduct problems (Bell *et al.*, 2018; Laurens *et al.*, 2017; Wildeman and Turney, 2014b). Additionally, there is a need for more evidence in relation to suicide events, with emerging evidence of an increased risk of suicide attempts, suicidal ideation and self-harming behaviours for children of parents with a history of contact with the justice system (Davis and Shlafer, 2017; O’hare *et al.*, 2022).

The gap in the research investigating the relationship between maternal incarceration and children’s rates of suicide and self-harm requiring hospital treatment warrants further investigation of these important outcomes. Recent estimates suggest that 54% of women entering prison in Australia have dependent children (Australian Institute of Health and Welfare, 2019). Children of incarcerated mothers have higher rates of child protection service (CPS) contact (Dowell *et al.*, 2018) and are more likely than other children to have a mother with mental illness (Burgess and Flynn, 2022; Fazel *et al.*, 2016). Both CPS contact and maternal mental illness are associated with an increased risk of offspring suicide and self-harm, and the risks for these exposures are increased by maternal incarceration (Hu *et al.*, 2017; Murray *et al.*, 2020). Incarceration rates for women have been increasing in recent years, both internationally (Walmsley 2018) and in Australia (Australian Bureau of Statistics, 2020). Parental incarceration is considered a key adverse childhood event (ACE) (Centers for Disease Control and Prevention, 2022; Metzler *et al.*, 2017), with ACEs long considered a crucial public health issue (Felitti *et al.*, 1998; Foege, 1998; Grimes 2017). Given that incarcerated mothers are more likely than incarcerated fathers to be the primary or sole caregiver to their children (Covington 2016; Glaze and Maruschak, 2008), maternal incarceration is an increasingly important public health issue that may have serious consequences for the well-being of their children. With suicide rates among 15–24 year olds in Australia increasing by 32% between 2010 and 2020 and with suicide the leading cause of death in this age group (Australian Institute of Health and Welfare, 2021), this issue has high policy relevance.

This study uses linked administrative data on children exposed to maternal incarceration and a non-exposed, matched comparison group to investigate the relationship between exposure to maternal incarceration and the risk of completed suicide and self-harm resulting in hospital service use. We aimed to (1) compare the rates of suicide and self-harm in children exposed to maternal incarceration and a matched comparison group; (2) investigate the role that related risk factors including CPS contact, maternal mental illness and living in a geographically remote location may play in the relationship between maternal incarceration and suicide or self-harm events; (3) investigate how suicide and self-harm rates change as individuals with and without a history of exposure to maternal incarceration move through different age groups (10–14, 15–19 and 20–28 years) to inform policymakers and practitioners when individuals may be most in need of targeted support or treatment.

## Methods

### Study design

This is a retrospective matched cohort study utilizing linked administrative justice, health and CPS data to compare the rates and risk of suicide and self-harm between individuals who were exposed to maternal incarceration prior to their 18th birthday and a non-exposed comparison group.

### Participants

The participants in this study were selected from a sample of children born in Western Australia (WA) between January 1, 1985, and December 31, 2011. Administrative records from the WA Department of Justice and Midwives Notification System (MNS) data collections were used to define the sampling frame for selecting participants. Our initial maternal incarceration-exposed and non-exposed groups contained 9,643 and 22,716 participants, respectively. The inclusion criteria for participants to be included in the exposed group were that they reached at least 10 years of age before the end of the study period and that they had to have a biological mother who was incarcerated at least once between conception (defined as 9 months prior to the child’s month of birth) and the month of their 18th birthday. Participants in the non-exposed comparison group also had to reach at least 10 years of age during follow-up and have a biological mother with no record of incarceration. Our outcomes of interest were death by suicide and hospital-treated self-harm in the child, occurring at age 10 or older. This is commonly used as the starting age in the existing literature for these outcomes (Ballesteros *et al.*, 2018; Morgan *et al.*, 2017), with evidence suggesting that suicide plans and attempts most frequently begin to emerge from age 10 onwards (Thompson *et al.*, 2012). After restricting the study to children who had reached at least 10 years of age during the study period, there were 7,674 children in the group exposed to maternal incarceration. These children were randomly matched 1:1 with children from the non-exposed group on sex, year of birth and Aboriginal status using the “ccmatch” function in Stata (StataCorp, 2019) as has been done elsewhere (Cook, 2015; Raj *et al.*, 2017). The non-exposed group included children whose mothers had no record of contact with the WA Department of Justice.

### Follow-up and observed outcomes

As we had month and year of birth rather than exact date of birth data for children, we used the 15th day of the month the child was born in as a pseudo “date of birth” for the purposes of analysis. Suicide and self-harm events were observed during the follow-up period, which commenced from the date the child turned 10 until death or the right censoring date, December 31, 2013.

### Data

The data for this study were linked through the WA Data Linkage System, using probabilistic matching and clerical review (Holman *et al.*, 1999). A list of all datasets, left and right censoring dates and which groups (exposed children, non-exposed children and mothers) the data pertained to is included in Table 1.

**Table 1.** Datasets included in the study

Dataset	Left–right censoring dates	Group(s) data pertain to
West Australian Registry of Births, Deaths and Marriages	May 1944**–**December 2013	Exposed and non-exposed children
		All mothers
Midwives Notification System	May 1944**–**December 2013	Exposed and non-exposed children
		All mothers
Department of Justice	April 1984**–**December 2013	Mothers of exposed children
West Australian Hospital Morbidity Data System	November 1984**–**December 2013	Exposed and non-exposed children
		All mothers
West Australian Emergency Department Data Collection	January 2002**–**December 2013	Exposed and non-exposed children
		All mothers
Mental Health Information System	January 1985**–**December 2013	All mothers
Department of Communities – Children Protection Service	November 1985**–**December 2013	Exposed and non-exposed children

### Demographic data

Demographic data recorded at birth included month and year of birth, child sex and geographic remoteness of the child’s home address (using the Accessibility/Remoteness Index of Australia [Hugo Centre for Population and Housing, 2020]) and were obtained from the MNS and Registry of Births Deaths and Marriages.

### Additional exposures

#### Maternal mental illness

Hospital Morbidity Data System (HMDS), Emergency Department Data Collection (EDDC) and Mental Health Information System data for mothers were used to ascertain maternal diagnoses of mental illness during the study period, using International Classification of Diseases, 9th revision, clinical modification (ICD-9-CM) (Medicode (Firm), 1996) codes 290–316 and International Classification of Disease, 10th revision, Australian Modification (ICD-10-AM) (National Centre for Classification in Health, 2004) codes F00–F99. Maternal mental illness was defined by any primary or additional diagnosis of mental illness in any of these datasets that occurred between the child’s conception and their 18th birthday. Using these data, a binary maternal mental illness exposure variable (yes/no) and a variable for the child’s age of incident exposure were created for each child.

#### Child protection service contacts

CPS data included notifications of a child abuse or neglect concern report made during the study period up until the child’s 18th birthday. Data included dates for all records, whether the notification was investigated, whether the investigation was completed, the outcome of the investigation and any action taken, which could include removal from the birth family into OOHC.

### Outcomes

#### Suicide

Date and cause of death were obtained from the WA Death Registrations dataset for participants who died during the study period. Suicides were identified in death data using ICD-9-CM codes E950–E959 for deaths up until June 1999 and ICD-10-AM codes X60–X84 for deaths from July 1999 to December 31, 2013.

#### Hospital-treated self-harm

Incidents of self-harm were ascertained from hospital admission and emergency department (ED) presentation data. HMDS data were obtained for the period 1985–2013 and included the dates of admission and separation from hospital as well as primary and contributory diagnoses (up to 20) and external causes (up to 4) for all inpatient episodes. EDDC data were extracted for the period 2002–2013 and included the presentation date, primary diagnosis, a single external cause and a “human intent of injury” variable that indicated whether, in the treating clinician’s opinion, an injury or poisoning was self-inflicted (Department of Health, 2020). All primary and contributory diagnoses and external causes were coded using ICD-9-CM (up until June 1999) and ICD-10-AM (from July 1999 onwards) codes. The same ICD codes used to identify suicide were used to identify hospital admissions for self-harm. An EDDC record was coded as self-harm if indicated by either one of the selected ICD codes or the human intent of injury variable.

### Statistical analysis

Median follow-up time was calculated for the exposed and non-exposed groups, then compared using a non-parametric *k*-sample equality-of-medians test. All socio-demographic data were binary variables except geographic remoteness, which used an ordinal scale and was recoded as a binary variable (metropolitan/inner regional versus outer regional/remote/very remote) for our analysis. Descriptive statistics were used to report socio-demographic and exposure characteristics (sex, Aboriginal status, geographic remoteness, CPS contact and exposure to maternal mental illness). Crude comparisons between groups were performed using chi-square tests for socio-demographic variables, CPS contact and maternal mental illness.

To achieve our first two aims, we used univariate and multiple Cox proportional hazards regression (Royston and Parmar, 2002) to analyse suicide (single event) and self-harm (repeated events, using an Andersen–Gill model [Andersen and Gill, 1982]) events and compare the unadjusted and adjusted risk, respectively, of suicide and self-harm occurring for the exposed and non-exposed groups. The multiple regression models for each outcome included our primary exposure (maternal incarceration), geographical remoteness, CPS contact and maternal mental illness as covariates.

To take into account the fact that some incident exposures occurred after the commencement of follow-up, we coded maternal incarceration, maternal mental illness and CPS contact as time-varying variables. For each child, we ascertained exposure to maternal incarceration from 9 months before birth (conception, calculated as −0.75 years of age) to 18 years of age. We coded this variable so that exposure status could vary after the start of follow-up. All participants exposed prior to the age of 10 were classified as exposed during follow-up, those who were exposed between the ages of 10 and 18 were classified as non-exposed at the start of follow-up and had their observations split at the time of incident exposure to maternal incarceration to reflect this change. We followed exactly the same procedure for exposure to maternal mental illness. We followed a similar procedure for CPS contact, which we recoded as a three-level ordinal variable: (1) no CPS contact (the reference group); (2) CPS contact without OOHC or (3) CPS contact with OOHC. Participants were assigned the highest level of CPS contact exposure they had experienced prior to the start of follow-up at age 10 and had their level of exposure changed during follow-up if they experienced a higher level of exposure at any time before the age of 18 during follow-up. It was possible for participants to have their CPS contact status change up to two times during follow-up if they had no prior CPS contact at the commencement of follow-up.

To achieve our third aim to investigate age-specific rates of events occurring without prior maternal incarceration exposure (non-exposed) and after maternal incarceration exposure (exposed), we calculated suicide incidence rates and self-harm event rates, stratified by three age groups: 10–14, 15–19 and 20–28 years. All analyses were performed using Stata version 16 (Statacorp, 2019).

Ethics approval for this project was granted by the WA Department of Health Human Research Ethics Committee (2013/72), The University of Western Australia Human Research Ethics Committee (RA/4/1/8111), the West Australian Aboriginal Health Ethics Committee (543) and the University of South Australia Human Research Ethics Committee (0000032609).

## Results

Of the 15,348 participants in the study (7,674 in each group) 7,466 (49%) were female and 9,724 (63%) were Aboriginal (Table 2). Total follow-up time was 142,410 person-years; median follow-up time was 9.2 years (IQR: 4.9–13.5) across all participants and did not differ significantly between the exposed and non-exposed groups (*P* = 0.923). There was a significant difference in CPS contact between the exposed and non-exposed groups. The exposed group was more than 13 times more likely than the non-exposed group to have had CPS contact with OOHC (27% vs. 2%, *P* < 0.001) and almost three times more likely to have CPS contact without OOHC (29% vs. 10%, *P* < 0.001). The exposed group was also almost three times more likely than the non-exposed group to have a mother who received healthcare for a mental illness (62% vs. 22%, *P* < 0.001).

**Table 2.** Participant characteristics for children exposed and non-exposed to maternal incarceration between conception and 18 years of age

Variable	Non-exposed (*n* = 7,674)	Exposed (*n* = 7,674)	*P*	Total (*n* = 15,348)
Female (%)	3733 (49)	3733 (49)	1.000	7466 (49)
Aboriginal (%)	4862 (63)	4862 (63)	1.000	9724 (63)
Outer regional/remote/very remote (%)	3821 (50)	2774 (36)	<0.001	6595 (43)
CPS contact without OOHC	747 (10)	2197 (29)	<0.001	2944 (19)
CPS contact with OOHC	183 (2)	2092 (27)	<0.001	2275 (15)
Maternal mental illness (%)	1711 (22)	4793 (62)	<0.001	6504 (42)
Median follow-up years (IQR)*	9.3 (4.9−13.6)	9.2 (4.9−13.5)	0.923	9.2 (4.9−13.5)

Chi-squared analysis used to compare groups except for

* *k*-sample equality-of-medians test.

CPS: child protection services; OOHC: out of home care; IQR: interquartile range.

Across the total sample, there were 43 deaths from suicide at a rate of 30.2 per 100,000 person-years, with no significant difference in rate between the exposed and non-exposed groups (Table 3). Small cell counts meant that we were unable to report age-specific suicide counts and rates due to data confidentiality. In our unadjusted Cox regression analysis, none of the covariates were significantly associated with an increased risk of suicide, and no covariates were significantly associated with suicide in our adjusted analysis (Table 4).

**Table 3.** Suicide and self-harm counts and rates per 100,000 person-years, stratified by prior exposure to maternal incarceration and age group (for self-harm only)

	Exposed		Non-exposed				Total	
	Events	Event rate (95% CI)	Events	Event rate (95% CI)	Rate ratio	*P*	Events	Event rate (95% CI)
*Suicide*								
All ages	24	36.7 (24.6, 54.7)	19	24.7 (15.7, 38.7)	1.49 (0.78, 2.87)	0.200	43	30.2 (22.4, 40.7)
*Self-harm*								
10−14 years	115	402.5 (335.3, 483.3)	40	103.3 (75.8, 140.9)	3.90 (2.70, 5.73)	<0.001	155	230.4 (195.6, 269.7)
15−19 years	338	1509.8 (1357.1, 1679.7)	163	689.6 (591.5, 804.1)	2.19 (1.81, 2.66)	<0.001	501	1088.6 (995.4, 1188.2)
20−28 years	401	2767.7 (2509.6, 3052.3)	152	1039.8 (887.0, 1219.0)	2.66 (2.20, 3.23)	<0.001	553	1899.9 (1744.8, 2065.0)
Total	854	1304.9 (1220.3, 1395.4)	355	461.3 (415.7, 511.9)	2.83 (2.50, 3.21)	<0.001	1209	849.0 (801.8, 898.3)

**Table 4.** Unadjusted and adjusted Cox proportional hazards regression for suicide events

Suicide	HR (95% CI)	*P*	aHR (95% CI)	*P*
Exposed to maternal incarceration	1.26 (0.69, 2.31)	0.447	0.92 (0.43, 1.96)	0.835
Outer regional/remote/very remote	0.58 (0.23, 1.48)	0.257	0.58 (0.20, 1.66)	0.307
CPS contact (ref no CPS contact)				
Without OOHC	2.65 (0.94, 7.47)	0.065	2.70 (0.91, 7.98)	0.073
With OOHC	1.39 (0.37, 5.29)	0.627	1.36 (0.33, 5.70)	0.676
Maternal mental illness	1.18 (0.53, 2.64)	0.683	1.06 (0.43, 2.66)	0.894

CPS: child protection services; OOHC: out of home care.

**Figure 1.**
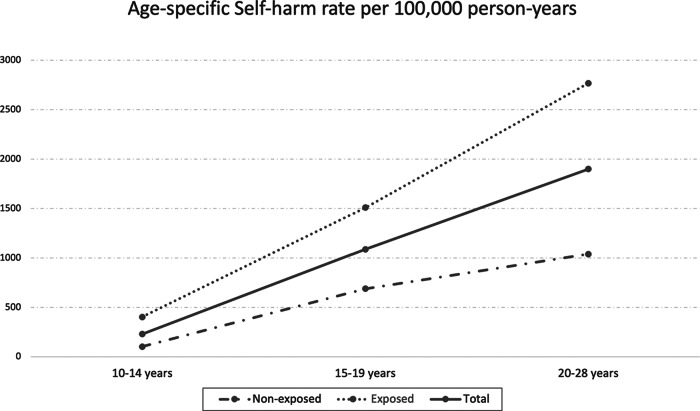
Age-specific self-harm rates for participants previously exposed and non-exposed to maternal incarceration.

There were 1209 hospital or ED-treated self-harm events during the study period, at an overall rate of 849.0 events per 100,000 person-years (Table 3). Overall, the exposed group (*n* = 854 self-harm events) had a higher rate of self-harm than the non-exposed group (*n* = 355 self-harm events, rate ratio [RR] = 2.83; 95% confidence interval [CI]: 2.50, 3.21). Among the age-specific self-harm rates (Fig. 1), the 20–28 years group had the highest rate overall at 1899.9 per 100,000 person-years. However, the biggest difference between those exposed and those not exposed to maternal incarceration was in the 10–14 years group, in which the rate of self-harm was significantly higher in the exposed group (RR = 3.90; 95% CI: 2.70, 5.73).

Our unadjusted Andersen–Gill Cox regression analyses showed that being exposed to maternal incarceration (HR = 2.84; 95% CI: 2.45, 3.29), having CPS contact without OOHC (HR = 4.02; 95% CI: 3.01, 5.35), having CPS contact with OOHC (HR = 5.99; 95% CI: 4.34, 8.26) and having a mother with a diagnosed mental illness (HR = 2.62; 95% CI: 2.15, 3.18) were all crudely associated with an increased risk of self-harm (Table 5). Conversely, being from an outer regional/remote/very remote area was negatively associated with self-harm (HR = 0.44; 95% CI: 0.36, 0.54). The results of our multivariable Andersen–Gill Cox regression analysis showed that after adjusting for covariates, the exposed group were at just under a 1.75 times higher risk of self-harm compared to the non-exposed group (adjusted hazard ratio [aHR] = 1.74; 95% CI: 1.45, 2.09) and that CPS contact without OOHC (aHR = 2.93; 95% CI: 2.16, 3.98) and CPS contact with OOHC (aHR = 3.06; 95% CI: 2.13, 4.37) were both independently associated with an increased risk of self-harm (Table 5).

**Table 5.** Unadjusted and adjusted Andersen–Gill Cox proportional hazards regression for self-harm events

Self-harm	HR (95% CI)	*P*	aHR (95% CI)	*P*
Exposed to maternal incarceration	2.84 (2.45, 3.29)	<0.001	1.74 (1.45, 2.09)	<0.001
Outer regional/remote/very remote	0.44 (0.36, 0.54)	<0.001	0.82 (0.64, 1.05)	0.117
CPS contact (ref no CPS contact)				
Without OOHC	4.02 (3.01, 5.35)	<0.001	2.93 (2.16, 3.98)	<0.001
With OOHC	5.99 (4.34, 8.26)	<0.001	3.06 (2.13, 4.37)	<0.001
Maternal mental illness	2.62 (2.15, 3.18)	<0.001	1.41 (1.12, 1.79)	0.004

CPS: child protection services; OOHC: out of home care.

## Discussion

This is the first study, of which we are aware, investigating both the relationship between experiencing maternal incarceration during childhood and rates and risk of suicide events and self-harm requiring treatment in hospital. We found that exposure to maternal incarceration during childhood was not associated with a higher risk of suicide; however, it was associated with a higher risk of self-harm, even after adjusting for maternal mental illness, CPS contact and remoteness. We also found evidence of an independent association between CPS contact and self-harm.

Our findings are consistent with previous research establishing a clear association between maternal incarceration and CPS contact (often resulting in OOHC) (Dowell *et al.*, 2018), high rates of mental illness experienced by mothers with a history of incarceration (Stanton and Rose, 2020) and a strong independent association between CPS contact and self-harm (Gnanamanickam *et al.*, 2022; Leckning *et al.*, 2021). Our findings are also broadly comparable to evidence of higher rates of self-harm in children of parents with criminal convictions (O’hare *et al.*, 2022). Our finding that there is a general increase in the rates of self-harm as people move through adolescence into adulthood is broadly consistent with Australian data from the general population covering a similar time period (Australian Institute of Health and Welfare, 2022b). However, our finding that the biggest difference in rates of self-harm between children exposed and non-exposed to maternal incarceration occurs during 10–14 years of age is novel and highlights a particularly vulnerable group.

As our study identifies, children exposed to maternal incarceration are a highly vulnerable group at increased risk of self-harm. This risk may continue once an episode of maternal incarceration has ended, as families experience ongoing challenges post-release. As maternal incarceration is a clear marker of severe familial adversity, service systems and structures need to carefully consider the needs of these children and their families. We suggest that ongoing holistic, multimodal and multilevel support for these children that incorporates support for their parents/carers and other family (Eddy *et al.*, 2019; Kjellstrand, 2017) is crucial to mitigate the risks these children face. It is also imperative that any policy response is underpinned by trauma-informed, age appropriate and culturally capable practice (especially given the over-representation of Aboriginal people in our sample and in the justice system generally [Australian Bureau of Statistics, 2021]) to reduce ongoing risks that contribute to poor mental health and self-harm for this group. Additionally, consideration should be given to expanding targeted support for children of incarcerated women (such as Shine for Kids in Australia and Person-Shaped Support in the United Kingdom [Dawson *et al.*, 2013]), along with the provision of family-friendly facilities where both mothers and children could meet. Providing this opportunity for mothers and children to maintain their connection in a suitable environment may help to prevent damage to the mother–child bond and foster and strengthen the relationship, benefitting both mother and child. Other possible intervention points to reach children exposed to maternal incarceration include school mental health services, contact with CPS and during ED presentation/hospital admission. To address the continuing risks that may be faced by these vulnerable children, it is crucial that support is initiated at the point of maternal incarceration and continued when a mother leaves prison.

Our results clearly show that children exposed to CPS contact, with or without removal to OOHC, were at considerably increased risk of self-harm, regardless of whether they had experienced maternal incarceration during childhood. Specific OOHC-related risks may flow from the deleterious effects of neglect and child abuse early in childhood (Berens and Nelson, 2015; Berger *et al.*, 2009), including high rates of complex and recurrent trauma experienced by children in the care system (Briggs *et al.*, 2012; Collin-Vézina *et al.*, 2011; Hanauer, 2015). For those who had experienced both maternal incarceration and CPS contact, additional factors may include the trauma associated with exposure to parental arrest, disruption to caregiving from the primary caregiver and challenging circumstances related to visiting parents in prison that may damage the mother–child bond (Arditti, 2012; Saunders and McArthur, 2013). For this group, these numerous, intersecting, often inter-related factors may combine in complex ways to contribute to an increased risk of children exposed to maternal incarceration engaging in self-harming behaviour. Given the high rates of CPS contact with OOHC we observed among children exposed to maternal incarceration, we support previous calls for research into the potential mechanisms for OOHC-related risks such as the quality of OOHC arrangements and the impact that disruptions to multiple aspects of children’s lives has (Berger *et al.*, 2009). Additionally, further research investigating how patterns of maltreatment and CPS contact may contribute to an increased risk of self-harm is needed to inform targeted intervention and prevention strategies for those most at risk.

Strengths of our study include the use of state-wide administrative data, which meant that we had population-level coverage and were able to investigate the risk of suicide and self-harm in all children and young people exposed to maternal incarceration in WA during the study period. Additionally, the use of linked administrative health and justice records helped to mitigate possible social desirability bias that may arise when relying solely on self or informant (e.g. parent or teacher) reported measures (Cherpitel *et al.*, 2018), particularly for sensitive and often stigmatized outcomes such as justice system involvement, self-harm and mental illness (Borschmann *et al.*, 2017). Additionally, being able to include CPS contact and other socio-demographic factors in our analyses enabled the examination of the contribution of these factors to study outcomes. Finally, the use of time-dependent covariates in our model allowed us to mitigate against the risk of immortal time bias and produce robust effect estimates.

One limitation of our study is the limited number of suicides recorded in our data, meaning that we were underpowered and limited in the type of analyses we could perform with respect to suicide. This may explain our finding of no significant difference in risk of suicide between our groups, which contrasts somewhat with previous research finding an association between experiencing parental incarceration and self-reported suicide attempts (Davis & Shlafer, 2017). Additionally, the follow-up period for our study ended when our oldest participant was 28, so our participants had not yet entered the age groups where the highest risk of suicide is observed in Australia (typically peaking around 50–54 years of age before sharp increases at around 80 years of age) (Australian Institute of Health and Welfare, 2022a), so it is possible that differences between groups could emerge later on. Future studies with larger samples and/or longer follow-up will be required to examine suicide in this population in greater detail. Another limitation of our study is that our data only capture self-harm events recorded in hospitals and EDs, so will likely be an underestimate of the true rates of self-harm. However, hospital data likely capture the vast majority of medically serious self-harm events. Finally, we did not have data on paternal incarceration and mental illness, which previous research has identified as an important factor in early childhood and psychological development (Whitten *et al.*, 2019, 2021).

## Conclusion

Children exposed to maternal incarceration are at a greater risk of self-harm compared to non-exposed children. Contact with CPS, with or without OOHC, was also independently associated with an increased risk of self-harm. Children who experience maternal incarceration should be provided with targeted holistic support that continues after maternal release from prison to reduce their risk of poor mental health and self-harm.

## Data Availability

This study was conducted using linked administrative data relating to individual study participants. These data were provided to the authors by multiple Government Departments subject to strict confidentiality requirements that prevent the authors from making the data publicly available.
